# Genetic Diversity of Aquatic *Ranunculus* (*Batrachium*, Ranunculaceae) in One River Basin Caused by Hybridization

**DOI:** 10.3390/plants9111455

**Published:** 2020-10-28

**Authors:** Jurgita Butkuvienė, Zofija Sinkevičienė, Donatas Naugžemys, Donatas Žvingila, Audrius Skridaila, Alexander A. Bobrov

**Affiliations:** 1 Life Sciences Center, Vilnius University, Saulėtekio av. 7, LT-10222 Vilnius, Lithuania; donatas.zvingila@gf.vu.lt; 2 Botanical Garden, Vilnius University, Kairėnų Str. 43, 01100 Vilnius, Lithuania; genetikas@gmail.com (D.N.); audrius.skridaila@bs.vu.lt (A.S.); 3 Nature Research Centre, Institute of Botany, Žaliųjų Ežerų Str. 49, 12200 Vilnius, Lithuania; zofijasin@gmail.com; 4 Papanin Institute for Biology of Inland Waters RAS, Borok, Nekouz distr., 152742 Yaroslavl reg., Russia; lsd@ibiw.yaroslavl.ru

**Keywords:** aquatic plants, *Ranunculus* sect. *Batrachium*, hybridization, molecular identification

## Abstract

Aquatic *Ranunculus* (sect. *Batrachium*) include homophyllous and heterophyllous plants. The development of floating leaves may be induced by genetic mechanisms or/and environmental conditions and this fact complicates the morphologically based identification of species. DNA-based studies provide the opportunity to expand the knowledge of this complicated group. We studied heterophyllous *Ranunculus* with well-developed capillary and intermediate leaves and visually homophyllous plants with capillary leaves from a single river basin, with the aim to evaluate their genetic polymorphism and taxonomic status—whether the plants with well-developed and weakly expressed intermediate leaves belong to different forms (taxa) or if they just express morphological variation of one or two taxa in a specific, very variable river environment. The studied heterophyllous and homophyllous plants from different rivers showed high genetic differentiation and a low level of genetic diversity within these groups. The molecular analysis did not reveal any inter simple sequence repeat (ISSR) polymorphism associated with the development of intermediate leaves. An analysis of nuclear ribosomal internal transcribed spacers ITS1–2 sequences revealed several ribotypes, which indicated the genetic heterogeneity of studied plants and indirectly confirmed the hybrid origin of some of them. Sterile plants originated from crossing of *R. circinatus* and *R. penicillatus* were discovered in the Skroblus River; however, identification of the parental species was impeded by the polymorphism detected. For this reason, cytological studies were performed and allowed confirmation of the hybrid origin of these plants.

## 1. Introduction

Water crowfoots, *Ranunculus* (sect. *Batrachium*), are characterized as morphologically heterogeneous and sensitive to changes in the environment [1,2]. This evolutionary young group of aquatic buttercups is well known for its intricate taxonomy determined by phenotypic plasticity, polyploidy, interspecific hybridization, and persistence by different ways of propagation [1,3,4,5,6,7,8].

River *Ranunculus* sect. *Batrachium* forms are a very complicated group for identification. In particular, the *Ranunculus penicillatus* complex combines different allopolyploids and hybrids [1,4,5,9]. As maintained by the classic literature [1], three groups of plants were distinguished within this taxon by leaf morphology: (1) homophyllous plants that produce only submerged, long capillary leaves; (2) homophyllous plants that produce only submerged, rather rigid capillary leaves; and (3) heterophyllous plants that produce capillary, floating, and intermediate leaves. In a recent study of *Ranunculus* sect. *Batrachium* [7], the homophyllous plants with capillary leaves were treated as *Ranunculus pseudofluitans* (Syme) Newbould ex Baker and Foggitt with flaccid leaves and *R. vertumnus* (C.D.K. Cook) Luferov with rather rigid leaves; the heterophyllous plants were treated as *R. penicillatus* (Dumort.) Bab., with the note that this name should be applied to the amphidiploid species originating from the crossing of *R. fluitans* and *R. peltatus*. Heterophyllous *R. penicillatus* is distributed in eastern and western European rivers but remains insufficiently understood because of its morphological heterogeneity and presumably hybridogenous origin [1,4,5,7,8,9]. Prančl et al. [8] indicated that genome size is a good marker for distinguishing of the most traditionally recognized species, but problems arise with difficult taxa, such as *R. penicillatus*, which is a group of different allopolyploids or hybrids.

The distribution of *R. penicillatus* and its occurrence in the flowing waters of the Baltic region including Lithuania remain unclear. Tzvelev [10] recorded heterophyllous *R. penicillatus* only in Latvia; additionally, the authors in [9] recorded it in Estonia.

During studies of aquatic *Ranunculus* in Lithuanian rivers, the unusual plants from the Merkys River basin (Southeast Lithuania) received special attention. They differed by the occurrence of more or less developed intermediate leaves and the absence of true floating leaves. One population in the Skroblus River has been known since 1983 and randomly observed to date. *Ranunculus* plants from this river are characterized by the permanent occurrence of capillary and intermediate leaves during the flowering period; however, true floating leaves have never been observed. Plants with solitary and sometimes hardly visible intermediate leaves or with capillary leaves only are observed in other rivers of this basin, in particular in the Ula River. The specimens in herbaria with intermediate leaves usually were tentatively identified as *R. penicillatus*, whereas with capillary leaves, only as *R. pseudofluitans* or *R. fluitans*. Cook [1] described many fertile and sterile hybrids and he suggested that intermediate leaves are frequently developed by hybrid forms between homophyllous and heterophyllous species. It has been shown [4,5,6,7] that interspecific hybridization within *Ranunculus* sect. *Batrachium* can make margins among species very unclear. This issue hampers determination of taxa based solely on morphological characters. Molecular methods have been previously used for the characterization of other aquatic plant species known for their phenotypic plasticity and taxonomic uncertainty [5,8,11,12,13,14]. The data obtained from molecular research could simplify the identification of species and provide a better understanding of their genetic properties and origins. *Ranunculus* sect. *Batrachium* has previously been the subject of molecular research [4,5,6,15]; however, in Lithuania, this type of investigation was started only recently [16].

For this DNA-based and cytological study, we selected heterophyllous (with well-developed and weakly developed intermediate leaves) and homophyllous (with capillary leaves only) aquatic *Ranunculus* plants from the different Merkys basin rivers, with the aim to assess whether the plants with well-developed and weakly expressed intermediate leaves belong to the different forms (taxa) or if they just express morphological variation of one or two taxa in a specific, very variable river environment.

## 2. Results

### 2.1. Genetic Diversity Based on ISSR-PCR Analysis

Inter simple sequence repeat (ISSR) polymorphism was assessed for all 33 plants sampled. A total of 53 bands were identified, of which 39 were polymorphic. Our attempt to identify morphotype-specific ISSR bands with any of the 27 tested oligonucleotide primers was unsuccessful. Specific ISSR bands for heterophyllous (Skroblus and Ula rivers) and homophyllous (Gruda and Ula) plants were not identified. All plant groups (from Gruda, Skroblus, and Ula rivers) showed similar genetic diversity parameters (Table 1).

Three plant groups showed an identical proportion of polymorphic loci at the 5% level (Table 1). All plant groups exhibited a similar measure of band richness (Br), ranging from 1.19 (Ula) to 1.20 (Skroblus), and expected heterozygosity (He), ranging from 0.06 (Gruda) to 0.07 (Skroblus). The lowest value of Shannon’s information index was also found in Gruda (0.09), and the highest index was in Skroblus (0.12).

Wright’s fixation index showed very high genetic differentiation among the studied populations (Fst = 0.796). In pairwise comparisons, the highest value of genetic differentiation was found between Gruda and Ula plants (Fst = 0.846), whereas the lowest value was between Skroblus and Ula plants (Fst = 0.709). Genetic differentiation between Gruda and Skroblus plants was 0.834 (Fst = 0.834). 

We also used hierarchical analysis of molecular variance (AMOVA) to reveal possible differences between the different phenotypic groups (heterophylous and homophyllous) of plants from these populations (Fst = 0.02, *p* < 0.05). Hierarchical AMOVA showed non-significant genetic differentiation among groups of homophyllous and heterophyllous plants (PhiRT = 0.019; *p* = 0.3) (Table 2). The highest genetic differentiation was among the plant groups from different rivers (81%). Within the groups, the molecular variance component reached only 17% (Table 2).

### 2.2. Ribosomal DNA ITS Region and Plastid rpl32-trnL Region Analysis

After comparison of the 26 established ribosomal DNA internal transcribed spacer (ITS) region sequences, a 573 bp long alignment was obtained. Analysis of sequencing data revealed 37 variable and 536 conserved sites. The 30 sites were potentially parsimony informative in the entire dataset. Only one site was identified as a single base insertion/deletion (Table 3 (30 bp position)). A single nucleotide polymorphism was detected at alignment positions 202 (*R. penicillatus* (KR995528, U5, U9, U11, and U12), 444 (*Ranunculus circintatus* G6), 445 (KR995528, U5, U9, U11, and U12), and 476 (KR995528, U5, U9, U11, and U12).

On the basis of the polymorphism of the ITS region sequences, seven different *Ranunculus* ribotypes were differentiated. Three individuals from the Gruda River presented two different ribotypes (G1, G11 one ribotype, and G6 another one). Seven plants from the Skroblus River belonged to five different ribotypes. All plants from the Ula River had the same ribotype. The identified sequences are deposited in the GenBank^®^ database (accessions numbers MH924769–MH924781, MT015510–MT015513).

**Figure 1:**
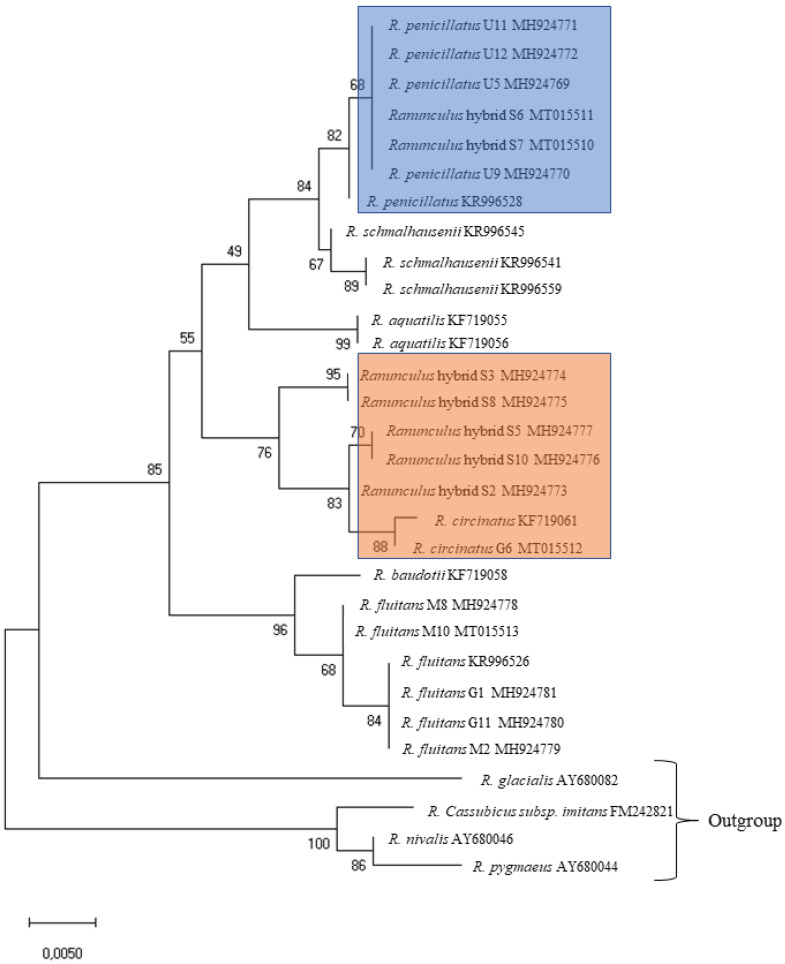
The ITS tree built using the maximum likelihood (ML) method showing relationships between 30 accessions of *Ranunculus* sect. *Batrachium*. Numbers indicate support values of ML analysis. Bootstrap support values were computed for 1000 replicates. The blue cluster combines *R. penicillatus* from the Ula River and *Ranunculus* hybrid sequences from the Skroblus River with the same ribotype of *R. penicillatus*; the orange cluster combines *R. circinatus* from the Gruda River and *Ranunculus* hybrid sequences from the Skroblus River with the same or very similar ribotypes of *R. circinatus* KF719061.

The sequences homologous for the obtained ITS sequences were searched in GenBank^®^, and 16 sequences were extracted. Individual variation and relation among some *Ranunculus* detected based on polymorphism of the ITS region is shown in the dendrogram (Figure 1).

Homophyllous plants with flaccid capillary leaves only with a few ultimate segments from the Gruda River (G1 (MH924781), G11 (MH924780)) showed the highest similarity to *Ranunculus fluitans* (Figure 1). These specimens shared one ribotype with samples from the GenBank^®^ database—M2 (MH924779) and *R. fluitans* (KR996526). Very high similarity to M2 was detected in the M8 (MH924778) and M10 (MT015513) individuals (Figure 1).

**Figure 2:**
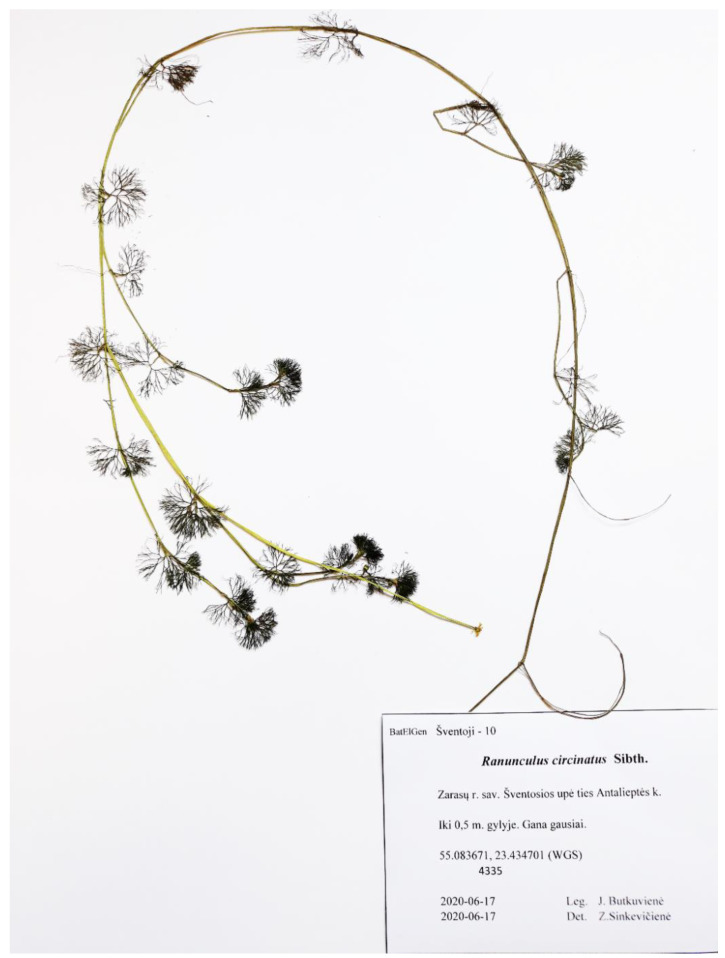
General appearance of *Ranunculus circinatus*.

Homophyllous plants with rigid capillary leaves only with numerous ultimate segments from the Gruda River showed the highest similarity to *R. circinatus* (Figure 2). The specimen G6 (accession number MT015512) shared a very similar ribotype with the sample of *R. circinatus* obtained from the GenBank^®^ database (KF719061) (Figure 1).

**Figure 3:**
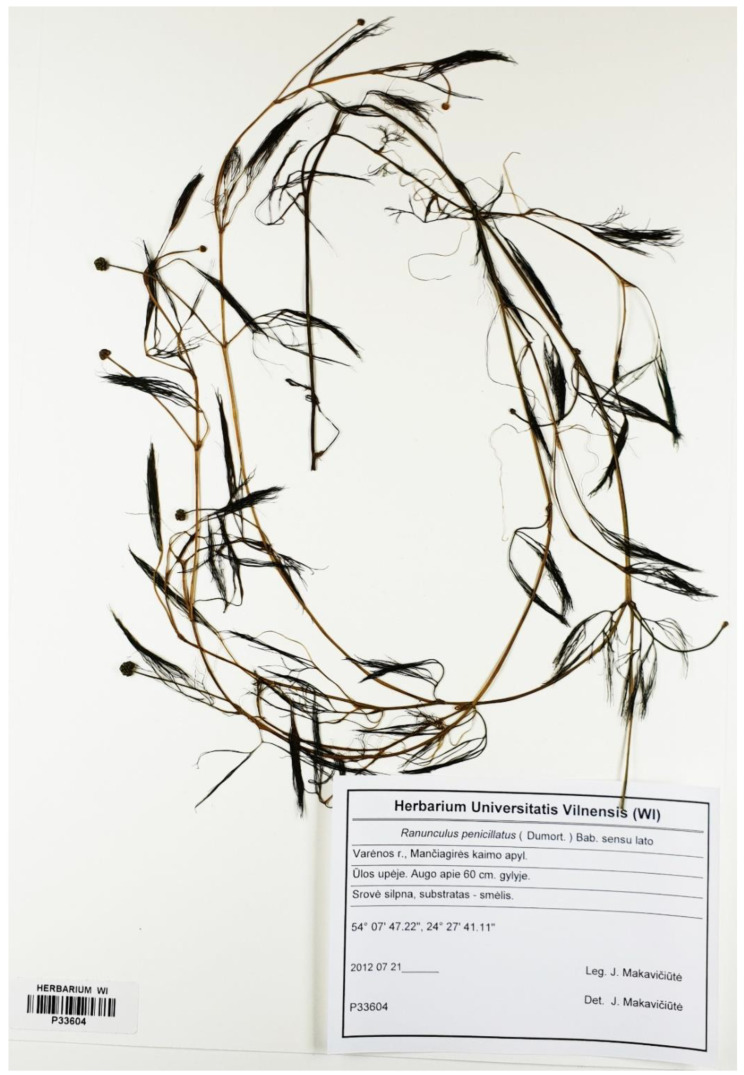
General appearance of putative *Ranunculus penicillatus* s.l. plant with capillary leaves only, from the Ula River.

**Figure 4:**
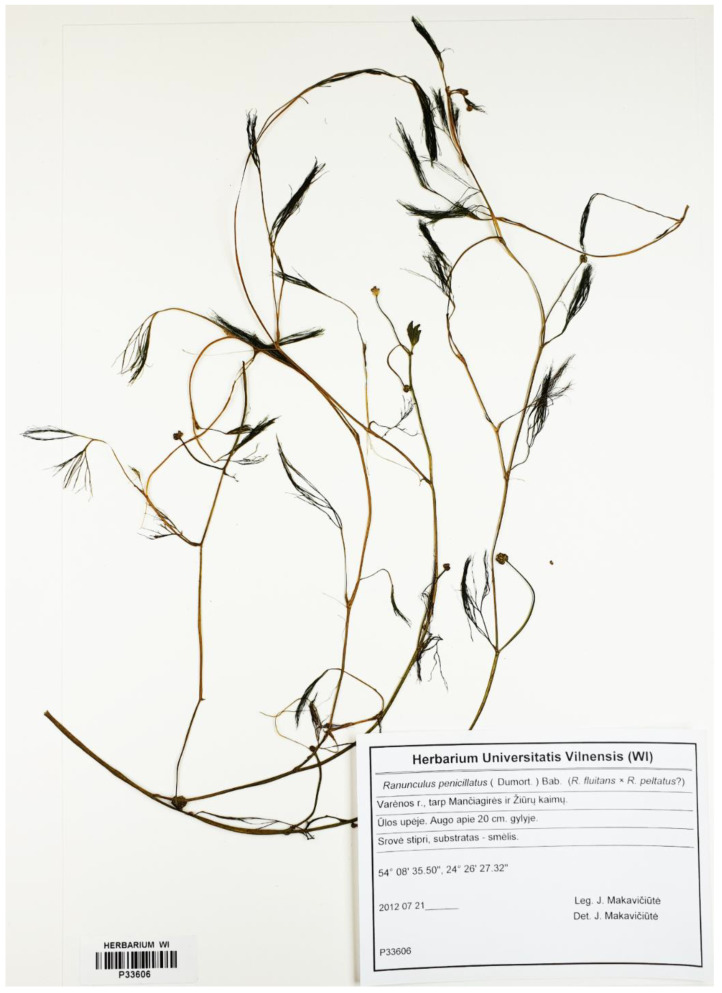
General appearance of putative *Ranunculus penicillatus* s.l. plant with capillary leaves and intermediate leaves from the Ula River.

In the Ula River, homophyllous individuals with flaccid capillary leaves with numerous ultimate segments (U9, U11, U12) were mostly presented (Figure 3) as well as one individual (U5) additionally with intermediate weakly developed leaves (Figure 4). Both kinds of plants were fertile (numerous heads with ripe fruits). All these specimens shared one ribotype (U5 (MH924769), U9 (MH924770). U11 (MH924771), U12 (MH924772)) and were very similar to a sample of *Ranunculus penicillatus* obtained from the GenBank^®^ database (KR996526). Based on morphological characters and molecular data, we treated these plants as *R. penicillatus*.

**Figure 5:**
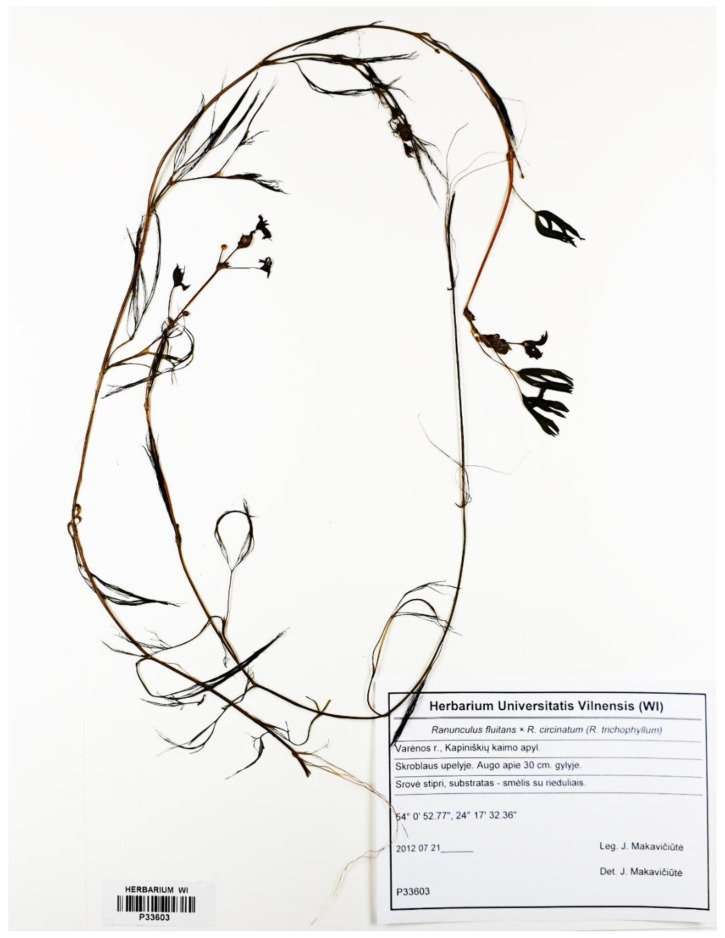
General appearance of putative *Ranunculus* hybrid with capillary and intermediate leaves from the Skroblus River.

In the Skroblus River, heterophyllous plants with developed intermediate leaves and flaccid capillary leaves with numerous ultimate segments occurred (Figure 5). The plants were sterile (weakly developed peduncles with empty heads). They exhibited five ribotypes (Table 3). The cluster analysis of the ITS region sequences of *Ranunculus* from the Skroblus sampling sites showed the highest similarity of sequences of the two ribotypes, S6 (MT015511) and S7 (MT015510), to sequences of *R. penicillatus* from the Ula River (U5, 9, 11, and 12 (MH924769–924772)) and was obtained from the GenBank^®^ database (KR996528), whereas sequences of the remaining three ribotypes clustered with sequences of *R. circinatus* from the Gruda River (G6 (MH924773)) and were obtained from the GenBank^®^ database (KF719061) (Figure 1).

Cluster analysis revealed that specimens with general morphology of *R. penicillatus* s.l. from the Ula and Skroblus rivers differ by their origin. Plants from the Ula River were fertile and they shared the same one ribotype similar to *R. penicillatus*. Plants from the Skroblus River were sterile and they shared sequences belonging to two groups, the first being similar to sequences of *R. circinatus* and the second to *R. penicillatus*. Sterility and such differentiation of the ITS sequences in the Skroblus River specimens suggest their hybrid origin.

**Figure 6:**
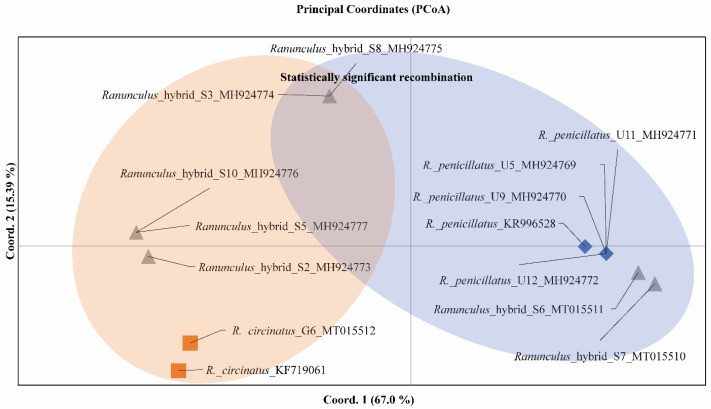
Principal coordinate analysis of supposed parental species (*R. circinatus* (orange) and *R. penicillatus* (blue)) and *Ranunculus* hybrid from the Skroblus River (grey).

PCoA was used to visualize the relationships among the supposed parental species and putative hybrid from Skroblus population. The two first coordinates described approximately 82.39% of the total genetic variability. Coordinate 1 explained 67.0% of the total variation and Coordinate 2 explained 15.39% (Figure 6).

The supposed parental species (*R. circinatus* and *R. penicillatus*) were grouped separately (Figure 6: orange zone—*R. circinatus*; blue zone—*R. penicillatus*). Principal coordinate analysis (PCoA) also revealed that hybrid plants from the Skroblus River were genetically differentiated. *Ranunculus* hybrids S2, S5, and S10 were closely located to *R. circinatus*, whereas, *Ranunculus* hybrids S6 and S7 were closely located to *R. penicillatus*. *Ranunculus* hybrids S3 and S8 were located distantly (Figure 6).

The maximum chi-square test (between two sequences and a putative derived sequence) showed that exactly *Ranunculus* hybrids S3 and S8 had statistically significant recombination after nucleotide 202 (Max Chi-squared = 10.4318, *p* < 0.0001). A statistically significant possible recombination was not detected in the remaining Skroblus individuals.

The alignment of cpDNA sequences determined from the cloned fragments that represented the full *rpl*32-*trn*L region was 966 bp long. Analysis of sequencing data revealed 15 variable and 951 conserved sites. All variable sites were identified as single nucleotide polymorphisms (SNPs). Additionally, single base pair insertion/deletion (indel) was identified in four individuals and 3-bp indel in three individuals. The analysis of this region did not show any tendencies of the connections of Skroblus hybrids and supposed parental species *R. circinatus* and *R. penicillatus* (i.e., maternal inheritance), because all sequences generally look similar.

### 2.3. Chromosome Numbers of R. circinatus, R. penicillatus, and Putative Hybrid

**Figure 7:**
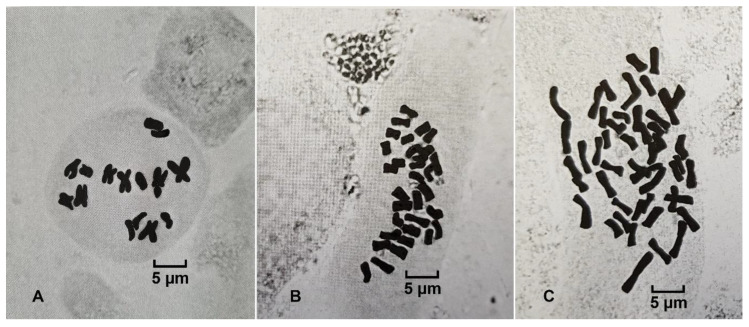
The metaphase plates of *R. circinatus* (2n = 16) from the Gruda River (**A**), putative *R. circinatus* × *R. penicillatus* (2n = 32) from the Skroblus River (**B**), and *R. penicillatus* (2n = 48) from the Ula River (**C**).

Karyological investigation demonstrated that specimens of putative hybrid *R. circinatus* × *R. penicillatus* from the Skroblus River were tetraploid (2n = 32), whereas the specimens of *R*. *circinatus* from the Gruda River were diploid (2n = 16) and *R. penicillatus* from the Ula River were hexaploid (2n = 48) (Figure 7).

## 3. Discussion

In this research, we tested the taxonomical belonging of river *Batrachium Ranunculus* plants with capillary leaves only (homophyllous) and differently expressed intermediate leaves (heterophyllous) occurring in three different rivers (Gruda, Skroblus, and Ula) of the same catchment area. Historically, before the last ice age, the Merkys River and its tributaries Gruda, Skroblus, and Ula formed one large river differentiated into separate rivers only after the ice age [17]. This event could be very important for the microevolution of aquatic plants. Thus, in the Skroblus River, *Stuckenia* × *fennica* (Hagstr.) Holub (*S. filiformis* (Pers.) Börner × *S. vaginata* (Turcz.) Holub) occurs without both parental species [18]. This is a well-known example of the occurrence of sterile hybrids in postglacial areas without parental species since the last glaciation [19]. Hybrid *Ranunculus* detected in this study grow in the Skroblus River together with *Stuckenia* × *fennica* and this could represent a similar case of sterile hybrids of ancient (glacial) origin.

The hybrid *Ranunculus* from the Skroblus River has a phenotype more similar to one parental species (typical *R. penicillatus*). However, typical heterophyllous *R. penicillatus* with developed floating, intermediate, and capillary leaves have never been recorded in Lithuanian rivers. However, except the Skroblus River, plants with only capillary or rarely capillary and weakly developed intermediate leaves treated as *R. penicillatus* (but never with “true” floating and clearly expressed intermediate leaves) often were found in the rivers of the Merkys basin [20]. Such a kind of morphological incongruence of the Skroblus hybrid phenotype and the Merkys basin *R. penicillatus* could be a result of morphological variation of the former caused by hybridization and specific river environment.

Inbreeding, cleistogamy, and clonal reproduction characteristics for *Batrachium* can decrease intrapopulation diversity [21] and could explain the low genetic diversity, but high genetic differentiation within the studied plants group. It is well known that small disjunct populations can be affected by genetic drift and inbreeding that also increase interpopulation differences [16,22,23]. Although, our study did not identify ISSR bands specific for different morphotypes and no significant molecular differences were detected by hierarchical AMOVA between groups of heterophyllous and homophyllous plants (*p* = 0.3).

The ITS region sequencing of heterophyllous hybrid plants sampled from the Skroblus River revealed five ribotypes that showed high similarity with the ribotypes of *R. circinatus* and *R. penicillatus* from other rivers and ITS regions of these species retrieved from GenBank^®^. These results could indicate that plants from the Skroblus arose from crossings of *R. penicillatus*, which according to [1] consists of allopolyploids originating from the hybrids of *R. fluitans* with *R. aquatilis*, *R. peltatus*, and *R. trichophyllus*, with rigid-leaved species *R*. *circinatus*. This was somehow unexpected because there were no clear morphological characters of *R. circinatus* in the Skroblus plants. In addition, the sequences of plants with intermediate leaves from the Skroblus River were not similar to those in a previous molecular analysis which allowed the detection of hybrid *R. fluitans* × *R. peltatus* from Poland [4]. Bobrov et al. [5] analyzed seven samples of *R. penicillatus* that did not show interindividual polymorphism in the ITS region. We identified this ribotype in the Ula River population with only differences in the absence of three polymorphic sites (positions 32 with A or G, 55 with C or T, 72 with C or T) and one transition of C instead of T at the 108 bp position. In the same publication of [5], five ribotypes were detected in *R. trichophyllus*. These ribotypes, considering the possible role of this species in the origin of *R. penicillatus* s.l., could explain the increase in ribotype polymorphism (five ribotypes) of aquatic *Ranunculus* from the Skroblus River. Additionally, it could arise as a result of multiple hybridization events, which implies not only phenotypic differences but also high differences at the molecular level [1,4,5,9]. The hybrid origin of *Ranunculus* from the Skroblus River from the lineage of *R. penicillatus* s. str. similar to the Ula River population and *R. circinatus*, indicated by genome differentiation and recombination, is confirmed by a maximum Chi-square recombination test.

The long formation of different genotypes could have been stabilized by clonal reproduction, but clonality may have an impact on the distribution of genetic diversity in natural populations [24]. The clonal reproduction may allow such sterile hybrids to form persistent populations and stabilize hybrid lineages [24,25]. Levels of genetic variability in sterile hybrids are determined by the amount of genetic diversity initially present in the parents, the frequency of hybrid formation events, the intensity of competitive exclusion among clones, the reduced effective population size in clonal populations, and somatic mutation [26,27]. Caetano-Anollés [27] notes that genome-wide mutation levels increase in vegetative culture. An accumulation of mutations within plants that are multiplying vegetatively at high rates could have important consequences for their biology and could be the cause of high genetic diversity [27,28,29]. Therefore, we also cannot exclude that besides recombination, additional sequence diversity of the sterile Skroblus individuals could have appeared from independent mutation events. This fact demonstrated again the complicated origin of the group of *R. penicillatus* and the interspecific hybridization during its evolution.

All obtained *rpl*32-*trn*L region sequences were similar. This is interesting because the rigid-leaved species, including *R. circinatus*, usually have very distinct sequences of plastid regions (e.g., [5]). This result can only be explained by the fact that the studied river population of *R. circinatus* can inherit non-characteristic plastid DNA as a trace of some hybridization in the past.

According to [8], polyploidy is frequent within aquatic *Ranunculus*, covering five ploidy levels ranging from diploids (2n = 16) to hexaploidy (2n = 48). Differences in ploidy level often have significant effects on phenotypic and reproductive traits [30]. Our study of chromosome numbers confirmed the hypothesis that *Ranunculus* with intermediate leaves from the Skroblus River (tetraploid) is a sterile hybrid between *R. penicillatus* (hexaploid) and *R. circinatus* (diploid). We may expect fertility to some extent in tetraploid hybrids, but, evidently, strong evolutionary differences between rigid-leaved species of the *R. circinatus* group with the remaining lineages of *Batrachium* caused sterility of this hybrid form [31].

## 4. Materials and Methods 

### 4.1. Plant Sampling 

**Figure 8:**
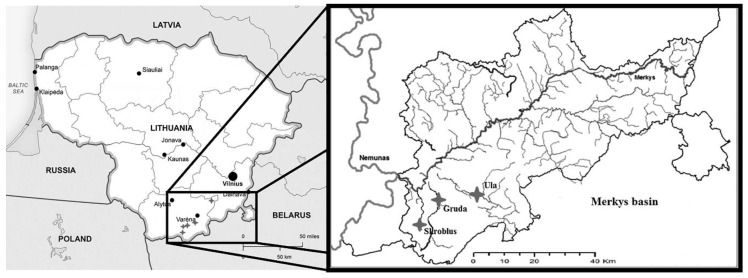
Map of the sampling sites from the Merkys River basin: Ula, Skroblus, and Gruda Rivers.

Aquatic *Ranunculus* plant were sampled from stretches of three different rivers (each ~100 m long) belonging to the Merkys River basin (southeast Lithuania): Gruda at 54°07′09.25″ N, 24°18′17.52″ E; Ula at 54°07′45.42″ N, 24°27′44.88″ E; Skroblus at 54°0′55.05″ N, 24°17′ 38.4″ E (Figure 8).

Samples were taken during July and August 2019 when plants are usually expected to have generative structures formed. A total of 33 samples of *Ranunculus* were collected and DNA was extracted for ISSR-PCR analysis: 11 plant samples from the Gruda River (G1–11), 10 from the Skroblus River (S1–10), and 12 from the Ula River (U1–12).

Eight samples of heterophyllous plants (with capillary and intermediate leaves) from the rivers Skroblus (S2, S3, S5, S6 S7, S8, S10) and Ula (U5) and 6 samples of homophyllous plants (with capillary leaves only) from the rivers Gruda (G1, G6, G11) and Ula (U9, U11, U12) were used for ITS and *rpl*32-*trn*L region sequencing.

### 4.2. DNA Extraction and ISSR-PCR Amplification 

Fresh plant leaves, 80–100 mg, were used for DNA isolation, according to a modified CTAB method [32]. The DNA amplification proceeded in a final volume of 10 µL, including 4 µL of DNA (5 ng/µL), 1.0 µL of 10× *Taq* buffer with KCl, 1.0 µL of dNTP mix (2 mM), 1.2 µL of MgCl_2_ (25 mM), 1.0 µL of a specific primer (1.0 OD unit) 0.08 µL of *Taq* polymerase (5 U/µL)) (Thermo Fisher Scientific Baltics, Vilnius, Lithuania), and 1.72 µL of deionized water (18.3 MΩ). ISSR-PCR was performed using a Mastercycler Personal (Eppendorf, Germany) thermocycler, as described earlier [16]: initial denaturation at 94 °C for 7 min; 32 cycles of: 94 °C for 30 s, specific temperature for each primer for 45 s, 72 °C for 2 min; ending with 72 °C for 10 min. In total, 27 different ISSR primers (ISSR I-28, ISSR I-39, Ward 1, Ward 2, ISSR I-34, ISSR I-50, Arcade 1, Arcade 4, ISSR-C, UBC 881, ISSR-E, ISSR-A, ISSR-D, ISSR-B, ISSR-G, ISSR-F, ISSR-H, ISSR-18, ISSR-32, ISSR-29, UBC807, UBC808, UBC810, UBC825, UBC827, UBC834 and UBC847) were tested; 10 primers (ISSR I-28 ((GT)_6_CG), ISSR I-39 ((AGC)_4_AC), Ward 1 ((AC)_8_T), Ward 2 ((AC)_8_G), ISSR I-34 ((AGC)_4_GG), ISSR I-50 (CCA(GCT)_4_), Arcade 1 ((CA)_8_GT), Arcade 4 ((GA)_8_TC), ISSR-C ((AG)_8_TG), UBC 881 (GGG(TGGG)_2_TGTG)) that produced clear and reproducible ISSR banding profiles were chosen for further analysis. The results of the amplification were visualized using 1.2% TBE–agarose gels stained with ethidium bromide. Images of the gel were made using the BioDocAnalyse (Biometra, Germany) system. Using the GeneRuler DNA LadderMix marker (Thermo Fisher Scientific Baltics, Lithuania), the bands in the gels were scored from 300 to 1800 bp. The reproducibility of amplified ISSR bands was examined by at least two independent repeats of PCR analysis and gel electrophoresis for all samples with all 10 primers. The comparison of banding pattern between two repeats resulted in 1245 phenotypic comparisons. The error rate was 1.1%. Only reproducible bands (1231) were included in the binary matrix and were used for analysis. The binomial matrix (1/0) of scored bands for further analysis was prepared, in which 1 indicated the presence of a band, and 0 indicated the absence.

### 4.3. ITS and rpl32-trnL Regions Sequencing 

Analyses of the ribosomal DNA ITS region (14 samples) and the plastid *rpl*32*-trn*L region (14 samples) were performed. Primers ITS1 (5′-TCCGTAGGTGAACCTGCGG-3′) and ITS4 (5′-TCCTCCGCTTATTGATATGC-3′) were used for the amplification of the nuclear DNA region [33]. The PCR was performed as follows: initial denaturation at 94 °C for 2 min; 30 cycles of: 94 °C for 1 min, 54 °C for 30 s, DNA synthesis at 72 °C for 2 min; ending with 72 °C for 5 min. Respectively, the primers *rpl*32 (5′-AGTTCCAAAAAAACGTACTTC-3′) and *trn*L (UAG) (5′-CTGCTTCCTAAGAGCAGCGT-3′) were used for the amplification of the non-coding chloroplast DNA region [34]. The PCR was performed as follows: initial denaturation at 80 °C for 5 min; 30 cycles of: 95 °C for 1 min, primer annealing at 50 °C for 1 min, DNA synthesis at followed by a ramp of 0.3 °C/s to 65 °C, and primer extension at 65 °C for 4 min; ending with 65 °C for 5 min. The preparation of amplified DNA fragments for sequencing was conducted as described earlier in [35]. Briefly, PCR products were visualized in a 1% TAE–agarose gel, and the bands were cut out and purified using a GeneJET Gel Extraction Kit (Thermo Fisher Scientific Baltics, Lithuania). To check the concentration and purity of the purified PCR products, a NanoDrop™ spectrophotometer was used (Thermo Fisher Scientific, Waltham, MA, USA). The purified DNA fragments were introduced to pTZ57R/T vectors using an InsTAclone PCR Cloning Kit (Thermo Fisher Scientific Baltics, Lithuania), following the manufacturer’s protocol. Plasmid DNA was isolated from 2–3 clones per sample using a Plasmid Miniprep DNA Purification Kit (Thermo Fisher Scientific Baltics, Lithuania) following the manufacturer’s protocol. The inserts were sequenced using primer M13/pUC (-46), 22-mer, and the reverse primer M13/pUC (-46), 24-mer, at the BaseClear B.V. (Leiden, The Netherlands) sequencing center. 

### 4.4. Cytological Analysis

Material for cytological analysis was collected in September 2019 from natural populations in the Gruda, Skroblus, and Ula rivers, where putative parental and hybrid plants occur. The tips of roots were taken from 10 plants from each river and 15 roots from each plant. In total, we analyzed 150 tips of roots of each taxon.

The root tips were pre-treated in a 0.1% colchicine solution for four hours and then fixed in a 3:1 mixture of 96% ethanol and acetic acid for two hours. The root tips were transferred into 70% ethanol. In the laboratory, root tips were stained by placing in 4% iron-ammonium alum for 10 min at room temperature and brought to a boil in 1% acetic hematoxylin twice. The squashed chromosome preparations of root tips were made with chloral hydrate (1:1) by using a standard root tip squash technique [36]. The metaphases were studied and photographed under an EVOS XL Digital inverted brightfield and phase contrast microscope with an image acquisition system.

### 4.5. Data Analyses 

For the ISSR dataset processing, Nei’s gene diversity (h) and Shannon’s information index (I) were calculated using the software PopGene [37]. Expected heterozygosity (He) and Wright’s fixation index (Fst) were estimated using the software AFLP-SURV 1.0 [38]. The band richness (Br) and proportion of polymorphic loci (PLP 5% level) using the rarefaction method for 8 individuals per population were calculated with AFLP-DIV v.1.1 [39]. 

Principal coordinate analysis (PCoA) was performed using the ITS dataset, and analysis of molecular variance (AMOVA) was performed using the ISSR dataset. Both analyses were performed with the software GenAlEx [40].

The MEGA X ver.10.1.8. program [41] was employed to align sequencing results with other *Batrachium* group ITS region and *rpl*32-*trn*L sequences from the NCBI GenBank database, which were extracted using the BLAST^®^ [42] tool. For analysis of the ITS region, the maximum likelihood (ML) method was performed with a Tamura 3-parameter model, which was selected based on the highest BIC (Bayesian Information Criterion) score. Bootstrap support values were computed for 1000 replicates.

For searching of recombinant sequences, the maximum Chi-square test (one derived and two parental sequences, significance test with 1000 replicates) for putative chimeras was implemented in START ver. 2 [43].

## 5. Conclusions

In conclusion, we did not reveal any evidence of ISSR polymorphisms associated with the development of intermediate leaves. The results of ITS analysis implied that genetic differences of the studied plants were produced by interspecific hybridization. Additionally, the studies revealed that heterophyllous plants from Skroblus River are an ancient, sterile hybrid between *R. circinatus* and *R. penicillatus*, which was not known to date [7], whereas the remaining homophyllous or almost homophyllous plants belong to fertile *R. circinatus*, *R. fluitans*, and *R. penicillatus*. Despite the fact that a new hybrid combination *R. circinatus* × *R. penicillatus* was discovered, it is impossible to make a formal description of a new nothotaxon because its morphological characters almost fully overlap with allopolyploid *R. penicillatus*.

The discovery of such an unusual sterile hybrid between diploid rigid-leaved species *R. circinatus* and allotetraploid *R. penicillatus* also support the point of view that network evolution by repeated hybridization events is one of the major mechanisms of speciation in *Batrachium* [7], especially in rivers and streams, which play a role of “evolutionary incubator” for such newly arising hybrids and polyploids [8].

## Figures and Tables

**Table 1:** The genetic diversity parameters of different *Ranunculus* sect. *Batrachium* populations. Band richness based on eight individuals (Br); polymorphic loci at the 5% level (PLP 5%); expected heterozygosity under Hardy–Weinberg genotypic proportions (He); gene diversity (h); Shannon’s information index (I); SD—standard deviation; SE—standard error.

Population	h ± SD	I ± SD	Br [8]	PLP 5%	He ± SE
Gruda	0.06 ± 0.13	0.09 ± 0.19	1.19	0.23	0.06 ± 0.02
Skroblus	0.07 ± 0.15	0.12 ± 0.22	1.20	0.23	0.07 ± 0.02
Ula	0.06 ± 0.14	0.10 ± 0.21	1.18	0.23	0.06 ± 0.02

**Table 2:** Analysis of molecular variance (AMOVA) for different groups of *Ranunculus* section *Batrachium*. Df—degrees of freedom; SS—sum of squared deviation; MS—mean squared deviation; Est.Var.—estimated variance; %—percentage.

Source	df	SS	MS	Est. Var.	%	Value	*p*
Among phenotypic groups	1	73.859	73.859	0.165	2%		
Among plants groups from different rivers	2	143.343	71.671	6.862	81%		
Within plants groups from different rivers	37	63.725	1.722	1.722	17%		
Total	40	280.927		8.749	100%		
PhiRT						0.019	0.298

**Table 3:**
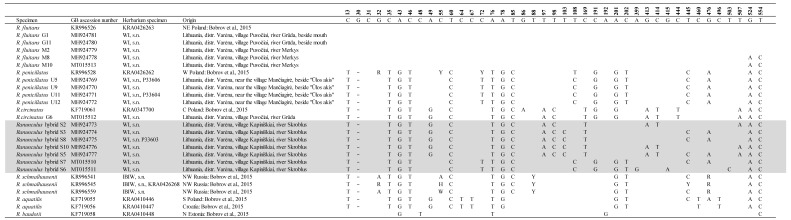
Sequence variation in the internal transcribed spacer (ITS) region of *Ranunculus* sect. *Batrachium* (14 sequences from populations Gruda, Skroblus (marked by grey color), and Ula and 12 sequences from the GenBank^®^ database). G—Gruda River (homophyllous plants); S—Skroblus River (heterophyllous plants); U—Ula River (homophyllous and heterophyllous plants).
